# Dermal wound transcriptomic responses to Infection with *Pseudomonas aeruginosa* versus *Klebsiella pneumoniae* in a rabbit ear wound model

**DOI:** 10.1186/1472-6890-14-20

**Published:** 2014-05-02

**Authors:** Kai P Leung, Peter D’Arpa, Akhil K Seth, Matthew R Geringer, Marti Jett, Wei Xu, Seok J Hong, Robert D Galiano, Tsute Chen, Thomas A Mustoe

**Affiliations:** 1 Microbiology Branch, US Army Dental and Trauma Research Detachment, Institute of Surgical Research, 3650 Chambers Pass, Building 3610, JBSA Fort Sam Houston, TX 78234, USA; 2 Systems and Integrative Biology, US Army Center for Environmental Health Research, Fort Detrick, Frederick, MD, USA; 3 Division of Plastic Surgery, Feinberg School of Medicine, Northwestern University, Chicago, IL 60611, USA; 4 The Forsyth Dental Institute, 245 First Street, Cambridge, MA 02142, USA

## Abstract

**Background:**

Bacterial infections of wounds impair healing and worsen scarring. We hypothesized that transcriptome analysis of wounds infected with *Klebsiella pneumoniae* (*K.p.*) or *Pseudomonas aeruginosa* (*P.a.*) would indicate host-responses associated with the worse healing of *P.a.-* than *K.p.-*infected wounds.

**Methods:**

Wounds created on post-operative day (POD) 0 were infected during the inflammatory phase of healing on POD3 and were harvested on POD4 for microarray and transcriptome analysis. Other wounds received topical antibiotic after infection for 24 hours to promote biofilm development, and were harvested on POD6 or POD12.

**Results:**

Wounds infected for 24 hours, relative to uninfected wounds, elevated transcripts of immune-response functions characteristic of infiltrating leukocytes. But *P.a.*-infected wounds elevated many more transcripts and to higher levels than *K.p.*-infected wounds. Coincidently, suppressed transcripts of both wounds enriched into stress-response pathways, including EIF2 signaling; however, this was more extensive for *P.a.*-infected wounds, including many-fold more transcripts enriching in the ‘cell death’ annotation, suggesting resident cutaneous cell toxicity in response to a more damaging *P.a.* inflammatory milieu. The POD6 wounds were colonized with biofilm but expressed magnitudes fewer immune-response transcripts with no stress-response enrichments. However, elevated transcripts of *P.a.*-infected wounds were inferred to be regulated by type I interferons, similar to a network unique to *P.a.*-infected wounds on POD4. On POD12, transcripts that were more elevated in *K.p.*-infected wounds suggested healing, while transcripts more elevated in *P.a.*-infected wounds indicated inflammation.

**Conclusions:**

An extensive inflammatory response of wounds was evident from upregulated transcripts 24 hours after infection with either bacterium, but the response was more intense for *P.a.*- than *K.p.*-infected wounds. Coincidently, more extensive down-regulated transcripts of *P.a.*-infected wounds indicated a stronger “integrated stress response” to the inflammatory milieu that tipped more toward cutaneous cell death. Unique to *P.a.*-infected wounds on POD4 and POD6 were networks inferred to be regulated by interferons, which may result from intracellular replication of *P.a.* These data point to specific downregulated transcripts of cells resident to the wound as well as upregulated transcripts characteristic of infiltrating leukocytes that could be useful markers of poorly healing wounds and indicators of wound-specific treatments for improving outcomes.

## Background

Wound healing proceeds in an orchestrated, highly ordered, and overlapping cascade of hemostasis, inflammation, proliferation, and remodeling [1,2] that can be deranged by local or systemic pathologies such as stress [3,4], diabetes [5-9], and infections [10-13], including biofilm infections [14,15], which can result in delayed wound closure and worsened scarring.

Microbial colonization of both acute and chronic wounds is inevitable. In many settings, gram-positive endogenous skin flora predominate [16]. While this is also the case for combat wounds [17], combat casualties hospitalized for definitive tertiary care in medical treatment facilities are at high risk for nosocomial infections [18,19] that develop days after injury and are largely due to multi-drug resistant gram-negative organisms including *Acinetobacter*, *Pseudomonas*, *Enterobacter*, and *Klebsiella*[18,20-22].

Biofilms are highly differentiated and spatially organized three-dimensional structures consisting of matrix-enclosed communities of one or more microbial species that form on colonizable surfaces [23]. Biofilms form rapidly in acute wounds [24,25] and contribute to the pathogenesis of chronic non-healing wounds [26-28].

Inflammation is the normal response to tissue injury and microbial infection, and in proper degree is required for tissue repair and healing. However, persistent inflammation or uncontrolled inflammation such as observed in chronic and/or infected wounds results in poor wound outcomes due to delayed wound closure and hypertrophic scarring [29]. The extent of inflammation and its clearance in a timely fashion can influence the rate and quality of wound healing [30,31].

Differences in the host-response to pathogens have been associated with virulence mechanisms. *P. aeruginosa* is a common gram-negative bacterium of nosocomial and life-threatening infections of immuno-compromised patients [32]. It possesses many virulence factors such as exoproteases (e.g., elastase), siderophores, exotoxins, hydrogen cyanide, and pyocyanin to attack host defenses. *K. pneumoniae* account for a substantial percentage of nosocomial infections in neonates, patients undergoing respiratory therapies, and patients hospitalized in urology and burn wards, but less is known about its virulence in wound infection and healing. Factors such as MagA (responsible for the mucoviscosity that correlates with high serum resistance *in vivo*[33]), siderophore aerobactin [34], and cell surface-associated fimbriae [35] have been implicated in *K. pneumoniae* virulence.

Using a full-thickness dermal rabbit ear wound model, we previously demonstrated that bacterial wound infections transitioned from active planktonic-phase infections to biofilm-dominant infections that delayed wound closure (epithelialization) and granulation tissue in-growth [11-13]. The extent of wound healing impairment depended on the infecting bacterial species. *Pseudomonas aeruginosa (P.a.)* caused the most healing impairment, *Klebsiella pneumoniae (K.p*.) slightly delayed closure relative to uninfected wounds, and *Staphylococcus aureus* was intermediate between *P.a.* and *K.p.*[12]. These infections stimulated the expression of inflammatory cytokines such as interleukin 1-β (IL-1β) and tumor necrosis factor-α (TNF-α) to levels proportional to the wound healing impairment [12].

Global gene expression analysis using microarrays has been used to define events in tissue repair [1], such as those that differ between healing and non-healing venous leg ulcers [36], skin wounds and oral mucosa wounds (a “privileged” tissue that heals rapidly without scar) [37], as well as fetal wounds which heal without scar and postnatal wounds that scar [38].

Here, we have used microarray/transcriptome analyses of wounds infected with *P.a.* or *K.p.* to identify similarities and differences in the wound responses to these differently virulent bacteria. The findings contribute to understanding the molecular foundations of the impaired healing caused by bacterial infections.

## Methods

### Animals

Under an approved protocol by the Animal Care and Use Committee at Northwestern University, adult female New Zealand white rabbits (3–6 months, ~3 kg) were acclimated to standard housing and fed *ad libitum*. All animals were housed in individual cages under constant temperature and humidity with a 12-hour light–dark cycle.

### Bacterial strains and culture

*P.a.* strain PAO1 was obtained from the laboratory of Dr. Barbara H. Iglewski, University of Rochester Medical Center. *K.p.* strain BAMC 07–18 was kindly provided by LTC Clinton Murray of Brooke Army Medical Center, Fort Sam Houston. To prepare the bacteria for the wound infection, *P.a.* PAO1 and *K.p.* BAMC 07–18 were grown in Luria (LB) broth and tryptic soy broth (TSB), respectively. After overnight incubation at 37°C, 0.5-mL of the *P.a.* and *K.p.* cultures were transferred to 9.5-mL of fresh sterile LB and TSB, respectively, and incubated at 37°C until the cultures reached the log phase. Bacteria were harvested and washed in phosphate-buffered saline (PBS) once by centrifugation at 4,000 rpm for 15-minutes at 4°C. The resultant pellets were resuspended in PBS and adjusted to an optical density of 0.5 at the wavelength of 600-nm (OD_600_). An OD_600_ 0.5 was equivalent to 10^8^ CFU per mL, which was determined empirically for each strain of bacteria used. Ten-μL of bacteria suspension at OD_600_ 0.5 from each strain was used as the inoculum (approximately 1 × 10^6^ CFU per wound) for infecting the rabbit ear wounds.

### Wounding and infection

Wounding, bacterial infection, and biofilm formation were adopted from our previously published wound biofilm model [11-13]. Briefly, rabbits were anesthetized with an intramuscular injection of ketamine (22.5 mg/kg) and xylazine (3.5 mg/kg) mixture prior to surgery. Ears were shaved, sterilized with 70% ethanol, and injected intradermally with 1% lidocaine /1:100,000 epinephrine at the wound sites. A total of 18 rabbits were used. Four, 6-mm diameter, full-thickness dermal wounds were created in the central area of the ventral ear down to perichondrium (4 wounds per ear). To minimize variations in dermal thickness and blood flow to the wounds, distal and proximal areas of the ear were not used. After surgery, wounds were dressed with Tegaderm (3 M Health Care, St. Paul, MN), a semi-occlusive transparent film. Individual wounds were inoculated with 10^6^ bacteria on postoperative day (POD) 3 and redressed with Tegaderm. Bacteria were allowed to proliferate *in vivo* under the Tegaderm dressing. A topical antibiotic, Ciloxan ointment (Ciprofloxacin 0.3%, Alcon, Fort Worth, TX), was applied on POD4 to eliminate free-floating, planktonic-phase bacteria, leaving a predominate biofilm. To prevent seroma formation and re-growth of planktonic bacteria, an antimicrobial, absorbent dressing containing polyhexamethylene biguanide (Telfa AMD, Tyco Healthcare Group, Mansfield, MA) was applied to wounds on PODs 5, 6, and then every other day until harvest. Uninfected and infected wounds were equally treated with the antibiotics and dressings. All dressings were examined daily throughout the protocol.

### Harvesting of wounds

After euthanizing the animals by intracardiac euthasol injection, wounds were harvested for RNA extraction. All wounds were excised using a 7-mm biopsy punch (Acuderm inc., Fort Lauderdale, FL). Wounds from different study groups were harvested at POD 4 (prior to the application of local antibiotic), POD6, and POD12, and RNA was isolated for microarray and reverse transcription quantitative PCR (RT-qPCR) analyses.

### RNA extraction

For mRNA extraction, the dermal layer on the dorsal side of the ear wound was removed and the wound bed was punched out and samples of all wounds were frozen in RNAlater (Ambion, Austin, TX) at −80°C for molecular analysis. Wound samples were homogenized using a Mini-bead beater-8 (Biospec Products Inc, Bartlesville, OK) using Zirconia beads (2.0 mm diameter, Biospec Products Inc) in the presence of Trizol Reagent (Sigma-Aldrich, St. Louis, MO). Total RNA was isolated according to the manufacturer’s protocol. Contaminating genomic DNA during RNA preparation was removed using the Turbo DNA-free kit (Ambion, Austin, TX). In general, 1 μg of RNA was obtained from each wound. The quantity and quality (Absorbance 260/Absorbance 280 ratio, 28 s/18 s RNA ratio, RNA integrity number) of the extracted RNA was determined using a NanoDrop 2000C UV–vis Spectrophotometer (Thermo Scientific, Wilmington, DE) and the Agilent 2100 Bioanalyzer (Agilent Technologies, Santa Clara, CA), respectively.

### Microarray

RNA (1 μg each) was collected from 5 control (uninfected), 5 *K.p.*-infected, and 5 *P.a.*-infected wounds at each time point (POD4, 6, and 12; 45 samples total). Each RNA sample was labeled with the One-Color Quick-Amp Labeling Kit (Agilent Technologies) and hybridized to an Agilent custom rabbit microarray (Agilent Product # G2519F) containing 4 sets of 44,000 probes per slide. The microarray contains 43,803 rabbit probes representing a total of 19,938 unique rabbit sequences. All labeling and hybridization was performed at the University of Florida Interdisciplinary Center for Biotechnology Research according to standard Agilent recommended protocols.

### Data collection and analysis

Data preprocessing was performed using the Bioconductor Limma package. The background correction was performed using Normexp with offset value 50. Quantile normalization was then used to correct for the non-biological variations across arrays. Probes were filtered based on the criteria that at least 39 samples must have a signal level above background and negative controls. The signal intensity values were further normalized by subtracting (in log2 scale) the time zero sample mean (i.e., POD3, immediately after wounding). Differentially expressed genes were identified using routines implemented in the Limma package to fit linear models to the normalized expression values [39]. The variance used in the t-score calculation was corrected by an empirical Bayesian method for better estimation under small sample size. All *p*-values were corrected by the Benjamini-Hochberg procedure.

### Ingenuity pathway analysis

Ingenuity Pathway Analysis (IPA, Ingenuity Systems Inc.) was used to analyze enrichments of DEGs (infected vs. uninfected wounds, and *K.p.-* vs. *P.a.*-infected wounds) into functions, pathways and networks. Because IPA does not analyze rabbit gene identifiers (IDs), they were first converted to their human ortholog gene IDs. Out of the 19,879 sequences on the microarray, 13,481 unique sequences mapped to human IDs and were ‘Analysis-Ready’ in IPA (68%). Data were analyzed through the use of causal analysis approaches in IPA to infer upstream biological causes and probable downstream biological effects, using enrichment score (Fisher’s Exact Test *p*-value) to measure overlap between observed and predicted gene sets, and z-score to match observed and predicted up/down regulation patterns [40].

### Reverse-transcription qPCR

Subsets of RNAs were used for determining transcript levels of specific target genes by RT-qPCR. RNA was used to prepare cDNA using superscript II (Invitrogen) with 100-ng of random primers (Invitrogen). RT-qPCR analyses using SYBR green was performed utilizing an ABI prism 7000 sequence detection system (Applied Biosystems, Foster City, CA). Genes that were highly differentially regulated in either *K.p.* or *P.a.* infected wounds as shown in the microarray analyses were selected for validation by RT-qPCR. PCR primers were designed using the Primer 3 program (http://frodo.wi.mit.edu/). Expression of each gene was normalized to the level of the house keeping gene, glyceraldehyde 3-phosphate dehydrogenase (GAPDH), to get ΔCt. The 2^-ΔΔCt^ method was used to calculate gene expression.

## Results

**Figure 1:**
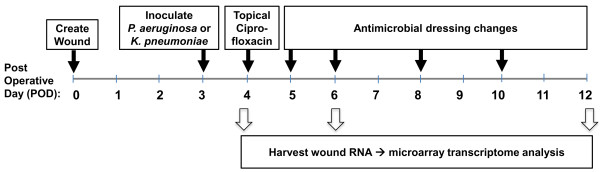
***Study design.*** On postoperative day 0 (POD0), 6-millimeter diameter wounds were created in the central area of the ventral ear down to the perichondrium. On POD3, wounds were inoculated with either *P. aeruginosa* or *K. pneumoniae* (10^6^ CFU/wound). On POD 4, topical ciprofloxacin ointment was applied. Antimicrobial absorbent dressing changes were made on PODs 5, 6 and every second day until wound harvest. Wounds were harvested on PODs 4, 6 and 12 by excision using a 7-millimeter biopsy punch, and RNA was isolated for microarray analysis.

To identify rabbit ear wound transcriptomic responses to infections with *K. pneumoniae* (*K.p.*) or *P. aeruginosa* (*P.a.*), we used the experimental design summarized in Figure 1. Six-mm diameter wounds were created and infected 3 days later (post-operative day three, POD3) with planktonically grown *K.p.* or *P.a.* (10^6^ colony-forming units, CFU). At three post-infection time points, wound RNA was harvested for microarray analysis. Wounds were first harvested the day after infection on POD4. Other wounds infected for 24 hours were treated with local antibiotic on POD4 to kill off planktonic bacteria. The RNA was isolated from these biofilm-infected wounds [12] two days later on POD6, and on POD12, when uninfected wounds were fully epithelialized, *K.p.*-infected wounds were slightly open (~1.7 mm epithelial gap [12]), and *P.a.*-infected wounds were largely open (~4 mm epithelial gap [12]). Gene expression levels of the infected wounds at each time point were compared with uninfected wounds, or the two types of infected wounds were compared each other, and the differentially expressed transcripts (DEGs) were analyzed for associations of with pathways, networks, and biological functions and disorders using the Ingenuity Pathway Analysis (IPA) database and software.

### Tallies of differentially expressed genes (DEGs) in infected vs. uninfected wounds

We found 1,378 differentially expressed sequences (fold change ≥ 2 with adjusted *p*-value ≤ 0.05) in *infected as compared to uninfected* (I/U) wounds out of the 19,879 unique sequences on the microarray; i.e., 7% of all gene probes were I/U differentially expressed (I/U-DEGs) by ≥2-fold, when tallying across all PODs for both *P.a.*- and *K.p.*- infected wounds (Table 1).

**Table 1:** **Summary of genes with >2-fold differential expression in *K.p. *- or *P.a. *-infected wounds vs. uninfected wounds**

	**POD4**		**POD6**		**POD12**	
Infecting bacteria:	***K.p.***	***P.a.***	***K.p.***	***P.a.***	***K.p.***	***P.a.***
Upregulated genes^*^:	168^^^	701	12	21	77	129
Downregulated genes^*^:	51	412	5	9	85	32
**Totals**	**219**	**1113**	**17**	**30**	**162**	**161**

^*^Based on ≥ 2 fold change of probe intensity with an adjusted *p*-value ≤ 0.05.

^^^Overlapping I/U-DEGs are included.

Averaging over all PODs, *P.a.*- infected wounds contained 3-fold more I/U-DEGs than *K.p.*-infected wounds. This was largely due to the 5-fold more I/U-DEGs in *P.a.*- than *K.p.*-infected wounds on POD4 the day after bacteria were inoculated into the wounds and before antibiotic treatment [12]. At this time exudate was abundant and the number of ≥2-fold-changed I/U-DEGs was 219 for *K.p.*-infected wounds and 1,113 for *P.a.*-infected wounds (Table 1). There were many fewer DEGs on POD6 (*K.p.*: 17; *P.a.*: 30) following two days of antibiotic treatment when ~1 to 2 logs fewer CFU were recovered than were inoculated (our previous study [12]). On POD12, the I/U-DEGs had increased (*K.p.*: 162; *P.a.*: 161, Table 1).

The complete list of all 19,879 gene probes and associated normalized log2 fold-changes, adjusted *p-*values, and updated annotations is provided in Additional file 1, and these data have been deposited in the NCBI GEO database (Accession number GSE51167).

### Few I/U-DEGs overlapped between PODs, but many I/U-DEGs were common to *K.p.*- and *P.a.*-infected wounds on each POD

**Figure 2:**
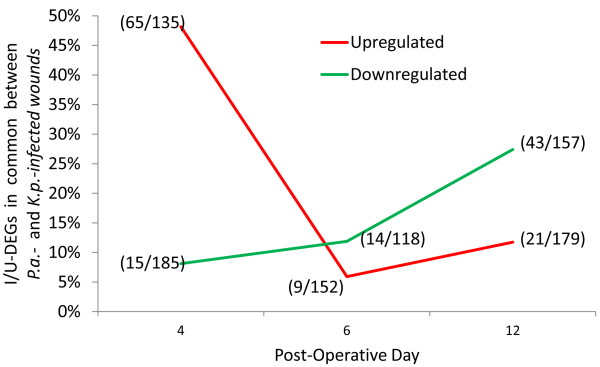
***I/U-DEGs in common between K.p.- and P.a.-infected wounds on POD4*.** Percent of Upr (red) and Dnr (green) I/U-DEGs in common between wounds infected by P.a. and K.p. on POD 4, 6 and 12 is plotted. The 100 most Upr and 100 most Dnr genes were analyzed for overlaps at each POD. Numbers in parentheses are the common/total I/U-DEGs between the two infected wound types. Fewer than 100 Upr and 100 Dnr I/U-DEGs were present on POD6.

To compare overlapping genes between PODs and infection types on each POD, we used the top 200 differentially expressed genes, consisting of the 100 most up-regulated (Upr) and 100 down-regulated (Dnr) I/U-DEGs, in order to compare equal numbers of I/U-DEGs. Not a single gene overlapped between all three PODs, for either species, and less than 7% overlapped between any two PODs, indicating the largely different states of infection and healing of the wounds on the three PODs. In contrast, many I/U-DEGs were common to *K.p.*- and *P.a.*-infected wounds (Figure 2) indicating similar host-responses to the different infections. To go beyond comparing similarities and differences of single genes, we analyzed similarities and differences of pathways and functions associated with the I/U-DEGs common and unique to the two infected wound types.

### POD4: Upr-I/U-DEGs common to *K.p.*- and *P.a.*-infected wounds enriched in inflammatory-response functions

The common I/U-DEGs to *K.p.*- and *P.a.*-infected wounds enriched in the IPA Function Annotation category ‘Inflammatory Response’. Downstream Effects Analysis of these common genes identified Functions Annotations whose activation state was inferred to increase, based on literature compiled in the Ingenuity® Knowledge Base, to include: ‘adhesion of immune cells’, ‘synthesis of reactive oxygen species’, ‘recruitment of neutrophils’, ‘activation of myeloid cells’, ‘activation of leukocytes’, ‘production of reactive oxygen species’, ‘adhesion of mononuclear leukocytes’, ‘activation of phagocytes’, ‘migration of neutrophils’, ‘cell movement of myeloid cells’, and ‘inflammatory response’. The Z-scores for enrichment of the I/U-DEGs in these functions were between 3.2 and 2.4 (significance cut-off 2.0), predicting an activated state. The top four inferred upstream regulators for these Upr I/U-DEGs common to both infected wound types were lipopolysaccharide (LPS), TNF, IFNG and IL1B (z-scores 3.5 to 2.5). These results show that the Upr I/U-DEGs common to the two infected wound types were mostly involved in the inflammatory response.

### POD4: 99% of I/U-DEGs were concordantly Upr or Dnr in *K.p.*- and *P.a.*-infected wounds

Seeking to identify differences between the host-responses to the two infected wound types, we mined more deeply into the dataset, analyzing all I/U-DEGs on POD4 with a *p*-value of ≤0.05 (Benjamini-Hochberg). The I/U-DEGs common to both *K.p.*- and *P.a.*-infected wounds summed to 907 (i.e., all overlapping I/U-DEGs). All of these I/U-DEGs, except for 1%, or 9, showed the same direction of differential expression in both infected wounds relative to uninfected wounds (45% Upr and 54% Dnr). This result suggests biological significance of even small I/U fold-changes and indicates largely similar responses of the wounds to the two types of bacterial infection.

The great majority of Upr-transcripts, 91%, were upregulated to a greater extent in *P.a.*-than *K.p.*-infected wounds. Many of these are mainly expressed in leukocytes, for example: the superoxide-producing NADPH oxidase expressed in neutrophils, NCF1, 2 and 4 (2.8-, 3.8- and 2.9-fold); the G-protein coupled receptor EMR2 (2.9-fold); S100A9 (1.9-fold); CSF3R (colony stimulating factor 3 receptor, granulocyte (2.9-fold); CSF2, colony stimulating factor 2, granulocyte-macrophage (5-fold); and CSF3, colony stimulating factor 3, granulocyte (3.6-fold). Conceptually, as compared to *K.p.*-infected wounds, some elevated transcripts of *P.a.*-infected wounds could come from greater numbers of infiltrating leukocytes. Similar numbers of leukocytes or cutaneous cells could have upregulated transcription to a greater extent in the absence of greater immune cell infiltration, but this seems less likely.

The majority of Dnr I/U-DEGs, 73%, were also downregulated to a greater extent in *P.a.*-infected wounds. More Dnr genes and a greater extent of downregulation of most overlapping I/U-DEGs in *P.a.*-infected wounds could represent a stronger stress response of cutaneous cells to greater inflammation caused by *P.a.* infection.

### POD4: Transcripts upregulated to a greater extent in *K.p.*-infected wounds appear to come from cutaneous cells responding to the inflammatory milieu

Of the 9%, or 36, of the concordantly Upr-transcripts that were upregulated to a greater extent in *K.p.*-infected wounds, only KRT8, CYR61 and CD5 were enriched in the function ‘inflammatory response’. However, KRT8 and CYR61 are likely not from leukocytes. KRT8 (keratin 8) is a type II intermediate filament that forms intermediate-sized filaments in the cytoplasm of epithelial cells [39]. And in fibroblasts, CYR61 appears to upregulate the expression of a number of genes involved in angiogenesis, inflammation, and matrix remodeling during wound healing [39]. The top ‘Physiological System Development and Function’ was ‘Connective Tissue Development and Function’ with seven enriching genes (CYR61, IQGAP1, NF2, KRT8, ALDH1A3, MXD1, and CYP27B1). Also another keratin, KRT78, was among the 36 genes. These enrichments suggest the genes more upregulated in *K.p.*-infected wounds came from cutaneous cells.

The second topmost ‘Molecular and Cellular Function’ into which the 36 Upr genes enriched was ‘Cell Death and Survival’. Eleven genes enriched in this function (CD5, AIFM3, CYR61, EGLN3, ALDH1A3, KRT8, MTFP1, MXD1, NF2, PDK1, and SLC5A8). The topmost Upr genes to a greater extent in *K.p.*- than *P.a.*-infected wounds were CYR61, EGLN3, and HILPDA. These three genes are all hypoxia inducible. Additionally, the top canonical pathway enrichment of the 36 genes was glycolysis.

The 36 genes that are Upr to a greater extent in *K.p.*-infected wounds are listed in Additional file 2. Their lack of extensive enrichment in inflammation functions and their enrichment in functions related to surviving the inflammatory environment suggest they represent the cutaneous cell response to the inflammatory milieu. Upstream regulators with a predicted activated state for regulating these 36 genes were only two: HIF1A and NUPR1. HIF1A is the master regulator of the response to hypoxia; under hypoxia it activates transcription of over 40 genes [41]. NUPR1 is as a component of the NUPR1/RELB/IER3 survival pathway that binds to chromatin to change gene expression that is associated with resistance to stress [41] and inhibition of apoptosis [42].

As an additional analysis, we directly compared differential expression between *K.p.*- and *P.a.*-infected wounds (i.e., K/P-DEGs), as opposed to the previous analyses of differential expression relative to uninfected wounds (i.e., I/U-DEGs). The K/P-DEGs that were more elevated in *K.p.*-infected wounds summed to 836 (*p*-value ≤0.05, Benjamini-Hochberg). The topmost four canonical pathway enrichments of these were ‘Cell Cycle Control of Chromosomal Replication’, ‘Cell Cycle: G2/M DNA Damage Checkpoint Regulation’, ‘Mitochondrial Dysfunction’, and ‘Role of BRCA2 in DNA Damage Response’. The topmost activated function was ‘cell survival’ (*p*-value 5.08E-03; 89 molecules; activation z-score 5.66). The topmost inhibited function was ‘organismal death’ (*p*-value 1.96E-03; 150 molecules; activation z-score -11.38). Similar results were obtained using the more limited set of K/P-DEGs with the cutoff of >2-fold change. None of the function enrichments were related to inflammation or response to infection. One possible explanation for this result is greater numbers of leukocytes had infiltrated *P.a.*-infected wounds, importing more inflammation transcripts than in *K.p.*-infected wounds. These data suggest that after 24 hours of active infection, cutaneous cells of *K.p.*-infected wounds activated pathways for surviving the particular conditions of the infection such as hypoxia and reactive oxygen species.

### POD4: Cutaneous cells are suggested to activate an “Integrated stress response”

Since the response to infection involves cells infiltrating the wound that only contribute transcripts to the wound, Dnr-transcripts come from cutaneous cells. The 27%, or 133, I/U-DEGs that were downregulated to a greater extent in *K.p.*- than *P.a.*-infected wounds had as their topmost enrichment in the category ‘Physiological System Development and Function’ the function annotation ‘Hair and Skin Development and Function’ (*p*-value 6.20E-06 to 1.95E-02; 7 molecules), additionally suggesting these transcripts come from cutaneous cells. These 133 I/U-DEGs’ topmost significant canonical pathway enrichments were ‘EIF2 Signaling’, ‘Regulation of eIF4 p70S6K Signaling’ and ‘mTOR Signaling’. Additionally, the topmost activated upstream regulator inferred for the 133 genes was the group N-cor (Activation z-score = 2.0), consisting of NCOR1 and NCOR2, nuclear receptor co-repressors that mediate basal transcriptional silencing [43]. In sum, these results suggest that cutaneous cells of *K.p.*-infected wounds activated an “integrated stress response” which involves downregulation of general translation and upregulation of specific transcripts to survive the stressors of *K.p.* infection [44,45].

The integrated stress response was also suggested for *P.a.*-infected wounds as the top three canonical pathway enrichments for 212 unique Dnr DEGs of *P.a.*-infected wounds were ‘Mitochondrial Dysfunction’, ‘Oxidative Phosphorylation’ and EIF2 signaling. These as well as ‘Regulation of eIF4 and p70S6K Signaling’ and ‘mTOR Signaling’ were also the top 5 canonical pathways into which enriched 85 Dnr-I/U-DEGs common to *K.p.*- and *P.a.*-infected wounds. Although these data indicate that the cutaneous cells of both types of infected wounds had activated an “integrated stress response” to survive the inflammatory milieu, the *P.a.*-unique I/U-DEGs enriched more into the ‘Cell Death’ function category. Using 212 unique Dnr-I/U-DEGs to each wound type, 17 from *K.p.*-infected wounds and 72 from *P.a.* -infected wounds enriched in the ‘Cell Death’ function annotation category.

### POD4: Discordantly regulated transcripts between *K.p.*- and *P.a.*-infected wounds

Of the 9 (1%) discordantly regulated I/U-DEGs between the two types of infected wounds, four were significantly Upr in *P.a.* and Dnr in *K.p.*: SPTAN1, ATP6V1F, ATG4A, and EEA1 (K/P-DEGs were 3.8-, 3-, 1.8- and 1.8-fold higher in *P.a.*, respectively). Each of these genes has a function related to intracellular organelles [41]. SPTAN1 encodes the filamentous cytoskeletal protein spectrin (alpha subunit, aka Fodrin), which functions as a membrane-stabilizing scaffold required for the function of intracellular organelles. ATP6V1F encodes a component of vacuolar ATPase (V-ATPase), which mediates acidification of eukaryotic intracellular organelles. ATG4A encodes a cysteine protease required for autophagy and is implicated in the processing of mature autophagosomes to facilitate their delivery to the lysosomal network. The EEA1-encoded protein binds phospholipid vesicles containing phosphatidylinositol 3-phosphate and participates in endosomal trafficking. Notably, *P.a.* virulence factor ExoS (expressed by PA01 in our *in vitro* experiments, unpublished observation) and in particular its ADP-ribosyltransferase activity, is required for intravacuolar replication of *P.a.* in epithelial cells [46].

The 5 I/U-DEGs that were elevated in *K.p.-* and suppressed in *P.a.*-infected wounds were LMO1, BRMS1L, CHD1L (K/P-DEGs were 2.5-, 1.4, and 1.5-fold higher in *K.p.*), as well as two transcribed loci EB374055 and DN885016. The three known genes encode chromatin binding proteins [41]. The LMO1-encoded protein is suggested to regulate transcription by competitively binding to specific DNA-binding transcription factors. BRMS1L encodes a component of histone deacetylase complexes. CHD1L encodes a DNA helicase that plays a role in chromatin-remodeling following DNA damage. One possibility is that *K.p.*-infected wounds retained capacity to remodel chromatin in response to the stress of the infectious inflammation.

### POD4: Network with upstream regulation by type I interferons unique to *P.a.*-infected wounds

Seeking to identify differences in upstream regulators associated with the gene expression patterns of the infected wound types, we analyzed upstream activating regulators of bacteria. They were markedly similar between the two wound types. Given the extensive overlap of identical and similar genes, to further search for discriminating features of the wound-response to *P.a.* and *K.p.* infections, we analyzed I/U-DEGs grouped by predicted activated upstream regulators (z-score ≥ 2) in the categories, ‘Cytokine’, ‘G-protein-coupled receptor’, ‘Growth factor’, ‘Ligand-dependent nuclear receptor’, ‘Transmembrane receptor’, and ‘Other’. The I/U-DEGs of each wound type without direct regulatory connections to other molecules in the same group, based on the IPA database, were excluded from the analysis. There were 89 upstream regulators of the 134 molecules common to both infected wound types, 69 upstream regulators of the 113 molecules unique to *P.a.*-infected wounds, and 3 predicted upstream regulators of the 66 molecules unique to *K.p.*-infected wounds.

Between the *K.p.*-*P.a.*-common and *P.a.*-unique I/U-DEGs, 44 upstream regulators overlapped. The remaining upstream regulators summed to 25 and are inferred to regulate 56 downstream I/U-DEGs unique to *P.a.*-infected wounds. Features of this network include regulation by type I and type II interferons, and 16 downstream molecular targets with more than four regulatory connections: INFG, STAT1, STAT4, PIM1, PIM2, CCL5, CSF2, MCL1, MMP1, MMP3, IRF1, IRF7, RSAD2, EGR1, DHX58, and OASL (Additional file 3). Although predicted to be activated by type I interferons, neither the type I interferons (IFNA, IFNA2, IFNA5, IFNL3, and IFNA14) nor the type I interferon receptor, IFNAR1, that were on our microarray were upregulated.

### POD6 wounds have fewer inflammation and stress-response transcripts, but Upr-transcripts from *P.a.*-infected wounds form a network with predicted upstream regulation by type I interferons as on POD4

By POD6, wounds infected for 24 hours and then treated with antibiotic for two days were likely colonized with biofilm, as approximately 10^5^ and 10^4^ CFU of *K.p.* and *P.a.*, respectively, were recovered from the wounds (our previous study [12]), which is 1 to 2 logs fewer than the 10^6^ CFU inoculated on POD3. Although about 10-fold fewer *P.a.* CFUs were recovered per wound, *P.a.* is known to replicate in vacuoles of epithelial cells [46]. Consistent with low bacterial burden as biofilm, both infected wound types had far fewer I/U-DEGs on POD6 than on POD4 (Table 1).

To assess similarities and differences in the response of the wounds to colonization with *K.p.* vs. *P.a.* biofilm, we analyzed enrichments of the common and the unique I/U-DEGs of *K.p.-* and *P.a.*-infected wounds. We analyzed the top fold-change I/U-DEGs, 100 Upr and 100 Dnr that were significant (adjusted *p*-value < 0.05), in order to compare nearly equal numbers of I/U-DEGs, since I/U-DEGs with >2 fold-change were so few. Neither *K.p.*-*P.a.*-common-I/U-DEGs nor the *K.p.*-unique I/U-DEGs enriched in functions with z-score >2.0. In contrast, the 94 I/U-DEGs unique to *P.a.*-infected wounds enriched in functions with predicted activation (z-score 2.7 to 2.0): ‘activation of T lymphocytes’, ‘antiviral response’, ‘activation of cells’, ‘activation of antigen presenting cells’, ‘cytotoxicity of lymphocytes’, ‘killing of cells’, ‘cell death of immune cells’, ‘activation of leukocytes’, ‘activation of cytotoxic T cells’, ‘antiviral response of cells’, ‘response of mononuclear leukocytes’, ‘fragmentation of DNA’, ‘cytotoxicity of T lymphocytes’, ‘quantity of cytotoxic T cells’, ‘maturation of phagocytes’, ‘maturation of antigen presenting cells’, ‘immune response of cells’, and ‘activation of macrophages’. While the 147 I/U-DEGs of *K.p.*-infected wounds barely made any regulatory connection amongst themselves, the 94 I/U-DEGs of *P.a.*-infected wounds connected into a 25-molecule regulatory network in which the major hubs were STAT1 and IRF7, with each having 16 direct regulatory connections. This 25-molecule network had several genes in common with the upstream-interferon-predicted network of I/U-DEGs unique to *P.a.*-infected wounds on POD4. These common genes between the POD4 and POD6 networks are STAT1, IRF7, and DHX58. Additionally, the POD6 network includes PSMB10, GBP1, and OAS2, while the POD4 network includes their highly related molecules PSMB9, GBP4, and OASL. These 25 molecules’ topmost five enrichments into canonical pathways are shown in Table 2. The molecule names, and the log ratios and *p*-value of their I/U-DEGs are listed in Additional file 4. Furthermore, the Upr I/U-DEGs enriched as their topmost network: ‘*Infectious Disease, Inflammatory Response, and Antimicrobial Response*’ (*p*-value 1x10^−47^ right tailed Fisher’s exact test). Table 3 lists the topmost five upstream mammalian receptors inferred to activate these I/U-DEGs. Although type I interferons are prominent among these, neither they nor their receptor were upregulated in our experiments.

**Table 2:** **Top five canonical pathway enrichments of 25 molecules from the network on POD6 unique to *P.a. *-infected wounds**

**Name**	***p*-value**	**Ratio**
Activation of IRF by Cytosolic Pattern Recognition Receptors	1.82E-08	5/72 (0.069)
Crosstalk between Dendritic Cells and Natural Killer Cells	1.11E-07	5/96 (0.052)
Role of Pattern Recognition Receptors in Recognition of Bacteria and Viruses	1.54E-07	5/106 (0.047)
Role of RIG1-like Receptors in Antiviral Innate Immunity	3.22E-07	4/49 (0.082)
Communication between Innate and Adaptive Immune Cells	6.08E-06	4/109 (0.037)

**Table 3:** **Topmost five upstream regulators predicted for the 55 Upr I/U-DEGs unique to *P.a. *-infected wounds on POD6 in the categories Cytokine, G-protein-coupled receptor, Growth factor, Ligand-dependent nuclear receptor, and Transmembrane receptor**

**Upstream regulator**	**Log ratio**	**Activation z-score**	**p-value of overlap**	**Target molecules in dataset**
TLR3	0.340	3.111	7.13E-19	CXCL10, DDX58, DHX58, GBP4, GCH1, IFIH1, IFIT1, IFIT3, IL15, IL23A, IRF7, STAT1, TLR3, TNFSF10, USP18, ZNFX1
IFN Beta		3.395	1.34E-16	CXCL10, IFIH1, IFIT1, IFIT1B, IL15, IRF7, STAT1, TAPBP, TLR3, TNFSF10, USP18, XAF1
Interferon alpha		3.913	1.67E-16	CXCL10, DDX58, DHX58, IFIH1, IFIT1, IFIT1B, IFIT3, IL15, IRF7, PRF1, STAT1, TAPBP, TLR3, TNFSF10, USP18, WARS
IFN type 1		2.087	3.44E-15	CXCL10, DDX58, DHX58, IFIH1, IFIT1, IFIT1B, STAT1, TNFSF10, UBA7
Ifn		2.793	4.54E-12	CXCL10, DDX58, DHX58, IFIH1, IFIT1, IL15, IRF7, STAT1, TLR3

Analysis of the Dnr I/U-DEGs for associations with upstream regulators and downstream functions found that despite the presence of upregulated inflammatory/antimicrobial transcripts, activation of stress-response pathways was not evident as on POD4.

### POD12: More inflammation and inhibited healing suggested for *P.a.*-infected wounds

On POD12, uninfected wounds had completely epithelialized, *K.p.*-infected wounds were nearly completely epithelialized (epithelial gap ~1.7 mm), and *P.a.*- infected wounds were more open (epithelial gap ~4 mm) [12]. In both *K.p.-* and *P.a.*-infected wounds, biofilm was seen on POD12 using scanning electron microscopy [12].

The I/U-DEGs common to *K.p.*- and *P.a.*-infected wounds (Upr and Dnr) enriched in the functions ‘Vasculogenesis’ and ‘Angiogenesis’, predicted to be activated with z-scores 2.2 and 2.0 (*p*-value 1.22E-02 and 1.97E-03, respectively). The top-scoring function was ‘Connective Tissue Disorders’ (*p*-values 1.27E-06 to 2.33E-03), which included ‘collagen type XI, alpha 1’, ‘collagen, type XII, alpha 1’, ‘collagen, type V, alpha 2’, ‘fibrillin 1’, ‘interleukin 18 binding protein’, ‘lysyl oxidase-like 2’, ‘matrix metallopeptidase 1 (interstitial collagenase)’, ‘thrombospondin 2’. All were downregulated except IL18BP and MMP1, which were upregulated.

We also analyzed *K.p.*- versus *P.a.*-infected wound differential expression (K/P). Transcripts significantly elevated in *P.a.*- relative to *K.p.*-infected wounds summed to 118. These molecules significantly enriched into ‘Inflammatory Response’, ‘Connective Tissue Disorders’, ‘Inflammatory Disease’, ‘Cellular Movement’, ‘Hematological System Development and Function’ and ‘Immune Cell Trafficking’ (movement and migration of phagocytes) as topmost functions. These 118 genes are shown in Additional file 5. Notably, the gene with the largest fold-change was PLK5, which was 10-fold elevated in *P.a.*- as compared to *K.p.*-infected wounds. PLK5 was upregulated in serum-starved fibroblasts as they exited the cell cycle, and PLK5 overexpression arrested cells at a G0/G1-like stage [47]. The topmost predicted upstream regulator for the 118 molecules elevated in *P.a.*-infected wounds was FGF1 (fibroblast growth factor 1, acidic), which trended toward an inhibited activation state (z-score -0.818; *p*-value 3.85E-04).

Conversely, molecules elevated in *K.p.*- relative to *P.a.*-infected wounds summed to 86 (Additional file 6). A single activating upstream regulator was the synthetic glucocorticoid methylprednisone (z-score 2.45, *p*-value 8.88E-03, 7 genes: SCAMP1, LDLR, HSD17B12, SDC2, CSNK2B, ATIC and FAH). Notably, endogenous glucocorticoid has been suggested to suppress granulation tissue formation, as mice with a DNA-binding-defective glucocorticoid receptor had early wounds with enlarged granulation tissue and a high fibroblast density [48]. PDGF BB was also a predicted upstream regulator, though it did not reach the activating z-score cutoff of 2.0 (z-score 1.41, *p*-value 1.22E-02, 5 genes: SLC7A7, MGST2, THBD, FMO1 and LDLR). Additionally, EVPL (Envoplakin) was 2.8-fold upregulated in *K.p.*-infected wounds over uninfected wounds and was 2-fold more highly expressed in *K.p.*- than *P.a.*-infected wounds. Envoplakin is a component of the keratinocyte cornified envelope, and is suggested to link it to desmosomes and intermediate filaments [39].

### q-RT-PCR validation of microarray results

Analysis of gene expression using q-RT-PCR of the same RNA samples as used for microarray analysis validated the microarray results (Additional file 7: Table S1). The selected genes represented those that were highly regulated either in *K.p.* or *P.a.* infected wounds. The qRT-PCR results confirmed the trend in expression of these selected genes revealed by microarray.

## Discussion

Wound gene expression patterns differed greatly between PODs 4, 6 and 12. This was expected as the wound states are different on these days, and respectively involve acute bacterial-induced inflammation, stabilization of biofilm, and healing. However, the wound responses to *K.p.* and *P.a.* infections showed similarities, especially on POD4. But there were significant differences. On POD4, the Upr gene expression patterns indicating inflammation were largely similar between the two infected wound types. But *P.a.*-infection induced a more intense response, with Dnr-transcripts indicating a stronger stress-response and the death of cutaneous cells. The greater inflammatory response to *P.a.* infection is likely associated with its pathogenic virulence that could have resulted in a larger bacterial load as compared to *K.p.* infection ([13] and unpublished observation). On POD6, after antibiotic killed the majority of planktonic bacteria and selected for biofilm, *P.a.*-infected wounds showed a network of inflammation/infection-response molecules predicted to be regulated by type I interferons, which was not associated with enrichments indicative of a stress response of cutaneous cells. The network of inflammation/infection-response molecules on POD6 was similar to a network unique to *P.a.*-infected wounds on POD4 that was minor part of the extensive inflammation/infection-response on that day. On POD12, *P.a.*-infected wounds showed indications of greater inflammation and impaired healing, which may be partly related to a larger *P.a.* biofilm load at this time as compared to *K.p.* biofilm, as suggested by our previous studies [13,49].

### POD4

#### Inflammation/infection-response

Seeking potential mechanisms of the very similar but much more extensive inflammatory host-response of *P.a.* vs. *K.p.* infection, we analyzed predicted upstream activating molecules. Those from bacteria were similar for both infected wound types. To further search for specific gene expression patterns that discriminate the two infected wound types, we analyzed other upstream regulators in categories of cytokines, growth factors and signaling receptors. Twenty-five upstream regulators, predominated by type I interferons, were inferred to regulate 56 I/U-DEGs unique to *P.a.*-infected wounds that enriched into the functions ‘Immune Cell Trafficking’ (36 molecules) and ‘Infection’ (39 molecules; *p*-values 1.24E-26 – 1.04E-7). In the ‘Infection’ category twice as many, 34, enriched into the subcategory ‘Viral Infection’ (*p*-value 2.56E-18) as enriched into ‘Bacterial Infection’ (17 molecules, *p*-value 8.37E-13). This network could contribute to upregulated IKBKE, which was upregulated 10-fold in *P.a.*-infected wounds but only 2-fold in *K.p.*-infected wounds. IKBKE is a non-canonical IkappB kinase with an important role in regulating antiviral signaling pathways. It is activated downstream of the recognition of LPS and nucleic acids by their PPRs—TLR3 (dsRNA), TLR4 (LPS), IFIH1 (long dsRNA), DDX58 (5’ppp ssRNA, short dsRNA), and ZBP1 (dsDNA) [50] — and activated IKBKE phosphorylates p65/RelA, interferon response factors 3 and 7 (IRF3 and IRF7), and STAT1. Consistent with a role in activating IKBKE, the network unique to *P.a.*-infected wounds on POD4 includes upregulated TLR3, IFIH1, DDX58, IRF3 and STAT1. The network unique to *P.a.*-infected wounds on POD4 and the similar network on POD6 could possibly be related to the intracellular replication of *P.a.*[46].

Some gene expression induced by *P.a.*-infection as compared to *K.p.*-infection probably involves known virulence mechanisms of *P.a.*[46,51], including the type III secretion effectors ExoS and ExoT that are expressed by the *P.a.* strain PAO1 that we used for our studies [52]. A previous study identified genes with induced expression dependent on ExoT activity [51] that are some of the same molecules that were highly expressed in *P.a.*- as compared to *K.p.*-infected wounds (e.g., ADM, DUSP5 and NR4A1). Additionally, many molecules targeted by ExoS activities (Ras, Ral, Rabs, and ERM proteins; as referenced in [53]) were differentially expressed in *P.a.*- vs. *K.p.*-infected wounds. For example, the ERM-like molecule NF2 was one of the 36 genes more upregulated in *K.p.*- than *P.a.*-infected wounds.

#### Stress-response

In parallel to the greater number of Upr-transcripts and their higher upregulation in *P.a.*-infected wounds on POD4, *P.a.*-infected wounds downregulated more transcripts, and most of the common Dnr-transcripts were downregulated to lower levels as compared to *K.p.*-infected wounds. Dnr-transcripts come from cutaneous cells, since cells that infiltrate the wound in response to infection only contribute transcripts. The Dnr-transcripts of infected wounds enriched in stress-response pathways and networks; these were several-fold more extensive in *P.a.*-infected wounds.

Given that a constant quantity of RNA was hybridized to each microarray, more leukocyte infiltration in *P.a.*-infected wounds would result in more leukocyte RNA and proportionally less fibroblast and keratinocyte RNA. But this does not appear to fully explain the greater extent of Dnr- transcripts in *P.a.* wounds on POD4 because *P.a.*-infected wounds had 4-fold more Upr-transcripts and twice as many, 8-fold, more Dnr-transcripts than *K.p.*-infected wounds (Table 1). And 26% of overlapping Dnr I/U-DEGs were more downregulated in *K.p.-* than *P.a.*-infected wounds. Also, stress responses are known to involve transcriptional and posttranscriptional processes including the specific stabilization and destabilization of mRNA as well as selective translation and transcription, and depend on the particular type of stress [54,55].

The downregulated transcripts assembled into networks with highly connected nodes such as CAND1 and cullins, which are involved in protein degradation, and the COP9 signalosome subunits. The COP9 signalosome functions in the regulation DNA repair, checkpoint control and chromatin remodeling [41]. DNA damage was a top activated function enrichment of Dnr I/U-DEGs unique to *P.a.*-infected wounds, which is a likely consequence of reactive oxygen species from the strong immune response to *P.a*. infection. The topmost predicted upstream regulator was MYC. MYC acts as a transcriptional amplifier, inducing expression of all genes to which it is bound [56]. The predicted inhibited state of MYC in *P.a.*-infected wounds is consistent with general suppression of transcription. Also upstream, PPAR (peroxisome proliferator-activated receptor) signaling was predicted to be in an inhibited state. PPAR signaling regulates many proteins in epidermis, which is very active in lipid metabolism, and where all PPAR and LXR (liver X receptor) isoforms are expressed, activating keratinocyte differentiation and the formation of the permeability barrier [57]. The mTOR pathway was also predicted to be in an inhibited state. mTOR is a conserved checkpoint protein that senses and integrates growth factor signals, and levels of nutrients, oxygen and energy. Major downstream targets of mTOR signaling are components of the translation machinery [58]. In contrast, RARgamma signaling was predicted to be activated, and RXRalpha-RARgamma heterodimers are necessary for growth arrest [59]. The Dnr-transcripts of *P.a.*-infected wounds enriched 4-fold more into the cell death function annotation than did an equal number of Dnr-transcripts of *K.p.*-infected wounds.

Previous studies have suggested that eIF2 signaling is activated during bacterial infections *in vitro*[60-63]. In epithelial cells, intracellular bacteria have been observed to up-regulate several proinflammatory cytokine genes (TNFA, IL1B and IL6) and chemokine genes (IL8 and CCL20) (as referenced in [63] and therein). These genes were among the most highly expressed genes in our *P.a.*-infected wounds. Also, human vaginal epithelial cells (HVECs) exposed to *Staphylococcus aureus* strongly upregulated chemokines and cytokines [64]. Among these, on our microarray, CCL20 was significantly upregulated only in *P.a.*-infected wounds (5-fold) and CXCL2 was upregulated 3- and 14-fold, respectively, in *K.p.-* and *P.a.-*infected wounds. However, our data cannot discriminate the source of these Upr-transcripts, leukocytes vs. cutaneous cells.

EIF2 signaling was among the top three canonical pathway enrichments for Dnr I/U-DEGs of both infected wounds. EIF2 is phosphorylated by several kinases in response to protein disequilibrium in the endoplasmic reticulum (ER) and cytoplasm, caused by diverse cellular stresses such as nutrient deprivation, exposure to the mTOR inhibitor rapamycin, high salt, redox perturbation, and/or DNA damage [65,66]. EIF2 phosphorylation shuts off global translation but allows synthesis of specific transcription factors and their target genes that support recovery from the stress (e.g., ER chaperones, oxidoreductases, and ERAD components). The central transcription factors in EIF2 signaling, ATF4 and ATF5, were not on our microarray, but the related transcription factors, ATF7IP, ATF6, ATF1, BATF2, ATF3, ATF2, and ATF7IP2 were upregulated in *P.a.*- relative to *K.p.*-infected wounds.

Beyond a threshold of stress, apoptosis can result. An apoptotic switch involving the Unfolded Protein Response (UPR) has been described [67]. The switch to the “terminal UPR” is thought to involve transcription of the pro-apoptotic transcription factor CHOP (DDIT3), which was upregulated 4.6-fold in *K.p.*-infected wounds and 13-fold in *P.a.*-infected wounds relative to uninfected wounds. CHOP has been suggested to induce death by promoting protein synthesis through increased transcription of GADD34 (PPP1R15A), which dephosphorylates EIF2 to reverse the inhibition of translation, exacerbating the preexisting ER stress [68,69]. Notably, in our study, GADD34 (PPP1R15A) was 30-fold upregulated in *P.a.*-infected wounds, relative to uninfected wounds, and was 7.5-fold more highly expressed in *P.a.*- than *K.p.*-infected wounds.

### POD6

On POD6, the wounds containing biofilm expressed magnitudes fewer inflammation transcripts than before antibiotic treatment. Although, 10-fold fewer *P.a.* than *K.p.* CFU were recovered from wounds on POD6 [12], only *P.a.*-infected wounds contained Upr I/U-DEGs that enriched in inflammation/infection functions. This network was virtually the only network found on POD6. It was similar to a network on POD4 consisting of Upr I/U-DEGs unique to *P.a.*-infected wounds, which was a minor part of the extensive inflammation/infection-response on POD4. In common between the two networks were three identical molecules and three highly similar molecules, and both networks contained molecules involved in nucleic acid sensing with inferred regulation by type I interferons. However, type I interferons were not upregulated on our microarrays. One possible explanation for these networks is that they are a consequence of growth of *P.a.* within epithelial cells [46] and could be regulated via an interferon-independent mechanism such as found in fibroblasts [70,71]. Another possibility is that these transcripts could come from a specific subpopulation of infiltrating cells such as dendritic cells [72]. Consistent with the greatly reduced inflammation on POD6, the Dnr I/U-DEGs of cutaneous cells did not significantly enrich into annotated functions suggesting a stress response.

Following two days of antibiotic treatment, POD6 wounds differentially expressed fewer genes than on POD4 or POD12. However, a large fraction of the few I/U-DEGs present in *P.a.*-infected wounds were related to each other through regulatory connections, including inferred upstream regulation by type I interferons. Such a network of transcripts could possibly be an identifying marker of *P.a.* biofilm colonization that has potential to develop into chronic infection that impairs healing.

### POD12

Relative to uninfected wounds, both types of infected wounds on POD12 differentially expressed genes that enriched into the functions ‘Vasculogenesis’ and ‘Angiogenesis’, with predicted activated states, and ‘Connective Tissue Disorders’. These similar functions between the two types of infected wounds are likely related to both being open as compared to the uninfected wounds that were closed on POD12.

Transcripts that were more highly expressed in *P.a.*- as compared to *K.p.*-infected wounds enriched in functions and networks related to the inflammatory response and connective tissue disorders, suggesting that *P.a.*-infected wound were more inflamed and disordered. Additionally, FGF1 (fibroblast growth factor 1) was the topmost upstream regulator of these molecules more highly expressed in *P.a.*-infected wounds. FGF family members possess broad mitogenic and cell survival activities, and FGF1 is a known modifier of endothelial cell migration and proliferation, as well as an angiogenic factor. However, as an upstream regulator of these DEGs, FGF1 was suggested to be in an inhibited state. Additionally, PLK5 (polo-like kinase 5) was upregulated 10-fold in *P.a.*- relative to *K.p.*-infected wounds. PLK5 has been shown to induced G1 arrest when overexpressed, to be downregulated in proliferating cells, and to accumulate in quiescent serum-starved fibroblast [73]. These results could indicate an impaired state of healing of *P.a.*-infected wounds. Conversely, upstream regulators of the more highly expressed transcripts in *K.p.*- vs. *P.a.*-infected wounds included a glucocorticoid and PDGF. These predicted upstream regulators may indicate lower inflammation (glucocorticoid) and active healing (PDGF) [74]. Overall these expression data and their regulator and function enrichments suggest that *P.a.*-infected wounds had greater inflammation and *K.p.*-infected wounds had more developed and active healing.

## Conclusions

These data contribute to defining the immune response of wound and proximal skin to bacterial infections of different virulence. In the clinical setting, diagnosis of infected wounds can be complicated by an absence of signs of inflammation (e.g., as on POD6) and late diagnosis can result in impaired healing [75,76]. Current diagnosis of infection is based on recoverable CFU which captures only culturable bacteria. PCR identifies more bacteria in wound samples, but mixed-species infections could have unpredictable virulence. The host-response counterbalances infections, and molecular markers of the host-response to infection may ultimately be useful for diagnosis and treatment. The current study suggests that patterns and extents of expression of Dnr-transcripts, expressed in cells resident to the wound and proximal skin, as well as specific Upr-transcripts, may contribute to the prediction or identification of poorly healing wounds, and may be useful for indicating wound-specific treatments to improve wound outcome.

### Ethics statement

The animal experiments were performed under animal protocol number 2010–1241 approved by Northwestern Medical School IACUC.

## Competing interest

KPL and MJ are employees of the U.S. Government. The work presented is part of their official duties. Title 17 U.S.C. §105 provides that ‘Copyright protection under this title is not available for any work of the United States Government.’ Title 17 U.S.C. §101 defined U.S. Government work as work by a military service member or employee of the U.S. Government as part of that person’s official duties. The opinions or assertions contained herein are the private views of these authors and are not to be construed as official or as reflecting the views of the Department of the Army or the Department of Defense.

## Authors’ contributions

Conceived and designed the experiments: KPL, TC and TAM. Performed the experiments: AKS, MRG, WX, SJH, and RDG. Analyzed the data: PD, KPL, TC, and WX. Wrote the paper: PD, KPL, and TC. Contributed reagents/materials/analysis tools and helped produce the final manuscript: MJ. Conceived of the study, obtained funding, participated in its design and coordination: KPL, TAM. Read and approved the final manuscript: KPL, PD, AKS, MRG, MJ, WX, SJH, RDG, TC, TAM.

## Pre-publication history

The pre-publication history for this paper can be accessed here:

http://www.biomedcentral.com/1472-6890/14/20/prepub

## Supplementary Material

Additional file 1 **List of gene probes and associated normalized log2 fold-changes and adjusted *p-*** **values.** Click here for file

Additional file 2 **Genes upregulated to a greater extent in *K.p.*-infected wounds on POD4.** Click here for file

Additional file 3 **Upstream regulators and the 56 downstream molecules unique to * P.a.*-infected wounds they are inferred to regulate on POD4.** Click here for file

Additional file 4 **Regulatory network of *P.a.*-infected wounds on POD6.** Click here for file

Additional file 5 **Transcripts elevated in *P.a.*- relative to *K.p.*-infected wounds on POD12.** Click here for file

Additional file 6 **86 transcripts elevated in *K.p.*- relative to *P.a.*-infected wounds on POD12.** Click here for file

Additional file 7: Table S1 RT-qPCR validation of microarray gene expression. Click here for file
