# Supplementary material for: Dermal wound transcriptomic responses to Infection with Pseudomonas aeruginosa versus Klebsiella pneumoniae in a rabbit ear wound model
Source: BMC Clin Pathol. 2014 May 2;14:20. doi: 10.1186/1472-6890-14-20 (PMC4101837; doi:10.1186/1472-6890-14-20)
Supplement: Additional file 7: Table S1 — RT-qPCR validation of microarray gene expression. [file 1472-6890-14-20-S7.ppt]

## Slide 1
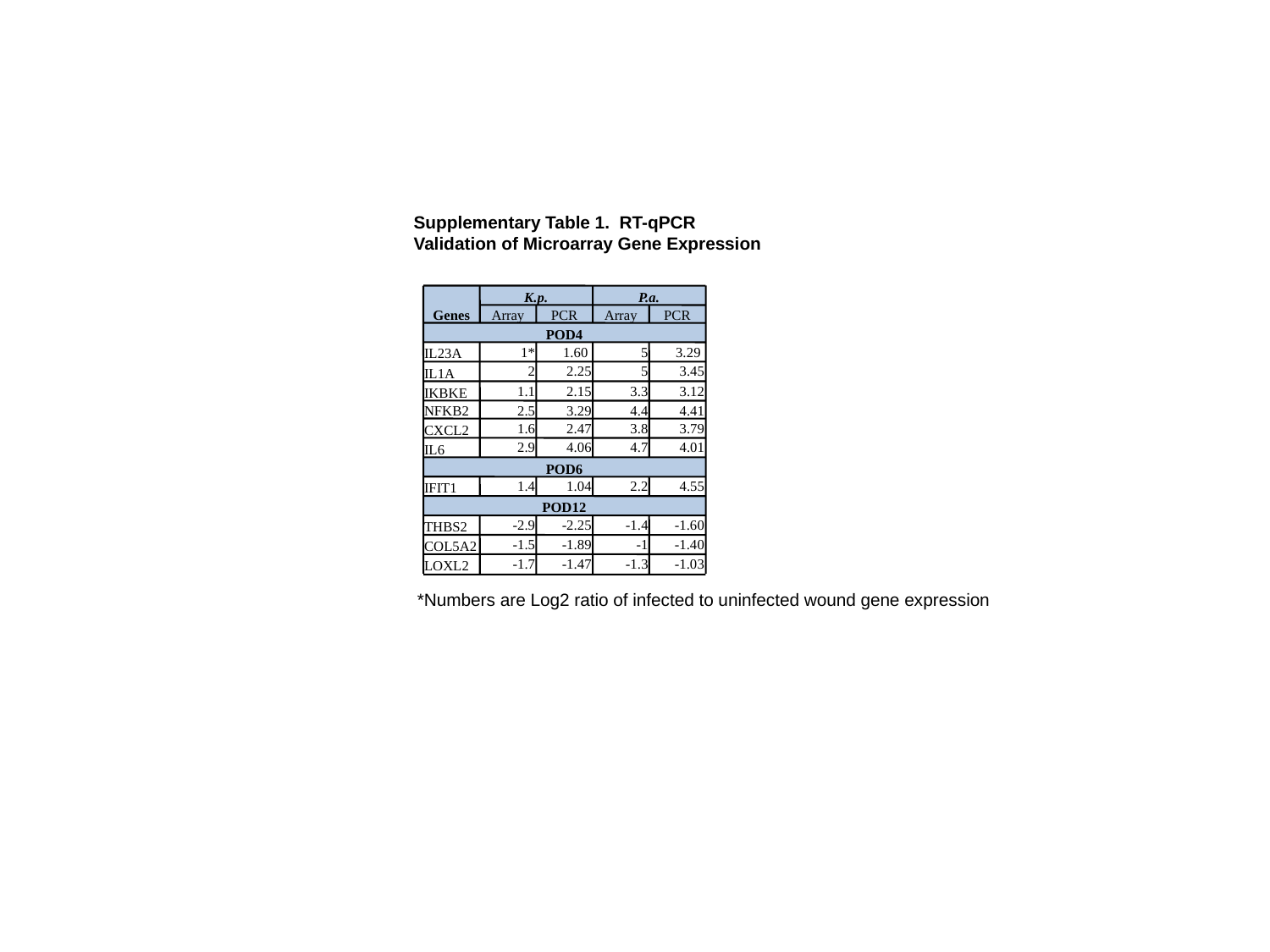

Supplementary Table 1. RT-qPCR Validation of Microarray Gene Expression
Genes
K.p.
P.a.
Array
PCR
Array
PCR
POD4
IL23A
1*
 1.60
5
3.29
IL1A
2
 2.25
5
 3.45
IKBKE
1.1
 2.15
3.3
 3.12
NFKB2
2.5
 3.29
4.4
4.41
CXCL2
1.6
 2.47
3.8
 3.79
IL6
2.9
 4.06
4.7
 4.01
POD6
IFIT1
1.4
 1.04
2.2
 4.55
POD12
THBS2
-2.9
 -2.25
-1.4
 -1.60
COL5A2
-1.5
 -1.89
-1
-1.40
LOXL2
-1.7
 -1.47
-1.3
 -1.03
*Numbers are Log2 ratio of infected to uninfected wound gene expression
